# Supplementary material for: Psychometric evaluation of the Asthma Diary Usability Questionnaire (ADU-Q): An adaptation and validation of a usability assessment tool among adults with Asthma
Source: PLoS One. 2026 Mar 9;21(3):e0343631. doi: 10.1371/journal.pone.0343631 (PMC12970881; doi:10.1371/journal.pone.0343631)
Supplement: S1 File — (DOCX) [file pone.0343631.s001.docx]

| Skor (score)  Domains and items | 1  Sangat tidak setuju *(Strongly disagree)* | 2  Tidak setuju  *(Disagree)* | 3  Agak tidak setuju  *(Somewhat disagree)* | 4  Neutral  *(Neutral)* | 5  Agak setuju  *(Somewhat agree)* | 6  Setuju  *(Agree)* | 7  Sangat setuju  *(Strongly agree)* |
| --- | --- | --- | --- | --- | --- | --- | --- |
| **Learnability** | | | | | | | |
| 1. Mudah untuk saya belajar cara  menggunakan diari asma ini  *(It is easy for me to learn how to use this asthma diary)* |  |  |  |  |  |  |  |
| 2. Diari asma ini mudah digunakan  *(The asthma diary is easy to use)* |  |  |  |  |  |  |  |
| 3. Susunan rangka diari asma ini mudah diikuti  *(The layout of the asthma diary is easy to follow)* |  |  |  |  |  |  |  |
| 4. Apabila saya melakukan kesilapan dalam merekod di dalam diari asma, saya boleh membetulkan tindakan saya dengan mudah  *(If I make a mistake in filling in the asthma diary, I can easily correct it)* |  |  |  |  |  |  |  |
| **Efficiency** | | | | | | | |
| 5. Format diari asma ini membenarkan saya merekod gejala asma saya dengan mudah  *(The format of the asthma diary allows me to record my symptoms easily)* |  |  |  |  |  |  |  |
| 6. Maklumat dalam diari asma teratur, membantu saya mencari maklumat yang diperlukan dengan mudah  *(The information in the asthma diary is well organized, helping me to look for information easily)* |  |  |  |  |  |  |  |
| 7. Jangka masa yang diperlukan untuk menggunakan diari asma ini sesuai dengan saya.  *(The duration of time required to use the asthma diary is appropriate for me)* |  |  |  |  |  |  |  |
| 8. Diari ini meningkatkan akses saya kepada perkhidmatan penjagaan kesihatan (contoh: memberi laporan gejala kepada doktor yang merawat)  *(The diary improves my access to healthcare service (e.g. reporting my symptoms to the attending doctor)* |  |  |  |  |  |  |  |
| 9. Diari asma ini membantu menguruskan kawalan asma saya dengan berkesan  *(The asthma diary helps to manage my asthma control effectively)* |  |  |  |  |  |  |  |
| **Satisfaction** | | | | | | | |
| 10. Saya suka format diari asma ini  *(I like the format of this asthma*  *diary)* |  |  |  |  |  |  |  |
| 11. Diari asma ini bermanfaat untuk kesihatan dan kesejahteraan saya  (*The asthma diary is beneficial for my health and well-being*) |  |  |  |  |  |  |  |
| 12. Diari asma ini mempunyai  semua fungsi dan kebolehan  yang saya harapkan (contoh:  merekod gejala asma di rumah)  (*The asthma diary has all that I*  *need for asthma self-*  *management e.g. document*  *asthma symptoms at home*) |  |  |  |  |  |  |  |
| 13. Diari asma ini adalah cara yang mudah diterima untuk mendapat penjagaan kesihatan yang baik (contoh: penilaian kesihatan diri)  (*The asthma diary is an*  *acceptable method to get*  *good self-care e.g. self-care*  *asssement*) |  |  |  |  |  |  |  |
| 14. Saya berasa selesa menggunakan diari asma ini dimana-mana sahaja walaupun bersama dengan orang lain seperti rakan-rakan dan keluarga  (*I feel comfortable using this asthma diary wherever I go even when around other people e.g. friends and family*) |  |  |  |  |  |  |  |
| 15. Saya akan menggunakan lagi  diari asma ini  (*I will use the asthma diary*  *again*) |  |  |  |  |  |  |  |
| 16. Secara keseluruhannya  saya suka diari asma ini  (*Overall, I like the asthma diary*) |  |  |  |  |  |  |  |
