# Supplementary material for: Psychometric evaluation of the Asthma Diary Usability Questionnaire (ADU-Q): An adaptation and validation of a usability assessment tool among adults with Asthma
Source: PLoS One. 2026 Mar 9;21(3):e0343631. doi: 10.1371/journal.pone.0343631 (PMC12970881; doi:10.1371/journal.pone.0343631)
Supplement: Attachment — Submitted filename: PONE-D-24-32929 -reviewed.pdf [file pone.0343631.s002.pdf]

PLOS ONE

The Adaptation and Validation of the Asthma Diary Usability Questionnaire (ADU-Q) in Adults with asthma

--Manuscript Draft--

**Manuscript Number:**	PONE-D-24-32929
**Article Type:**	Research Article
**Full Title:**	The Adaptation and Validation of the Asthma Diary Usability Questionnaire (ADU-Q) in Adults with asthma
**Short Title:**	Asthma Diary Usability Questionnaire (ADU-Q)
**Corresponding Author:**	Nik Munirah Nik Mohd Nasir<br>Universiti Teknologi MARA<br>Selayang, SELANGOR DARUL EHSAN MALAYSIA
**Keywords:**	asthma diary; Self-management; usability
**Abstract:**	The usability assessment of an asthma self-management tool is important to ensure its effectiveness; however, the availability of a validated tool is scarce. The aim of this study is to adapt, validate and assess the reliability of the Asthma Diary Usability Questionnaire (ADU-Q) to measure the usability of an asthma diary among adult patients with asthma. The study was conducted in 3 phases. The first phase was divided into adaptation and content validation stages. The adapted questionnaires underwent domain validation by 3 experts and item content validation by 7 experts. The second phase involved face validation with 10 asthma patients who attended the primary care and respiratory clinic. This is followed by construct validation and test-retest reliability. 115 asthma patients attending the primary care and respiratory clinic were recruited to answer the ADU-Q questionnaire and 62 patients were called back to fill in the same questionnaire after 2 weeks. The psychometric properties were determined using external factor analysis (EFA) with promax rotation. The reliability testing was performed using Cronbach's Alpha and the intraclass correlation coefficient (ICC). Each item in ADU-Q went through two rounds of domain and item validation. The calculated item content validity index (I-CVI) results were >0.9. The item face validity index (I-FVI) result ranged from 0.9 to 1.0. The EFA with 3 fixed factor solutions produced 7 items loaded to Factor 1, 4 items loaded to Factor 2 and 5 items loaded to Factor 3. The Cronbach Alpha for ADU-Q was 0.982, indicating good internal consistency and the ICC value was 0.988, indicating good test-retest reliability. Based on these findings, ADU-Q is a valid, reliable and stable tool for assessing the usability of an asthma diary among adults with asthma.
**Order of Authors:**	Sharifah Idayu Sayid Abdullah<br>Nik Munirah Nik Mohd Nasir<br>Lina Lohshini Kanoo<br>Nur Amirah Shibraumalisi
**Opposed Reviewers:**	
**Additional Information:**	
**Question**	**Response**
**Financial Disclosure**<br><br>Enter a financial disclosure statement that describes the sources of funding for the work included in this submission. Review the submission guidelines for detailed requirements. View published research articles from *PLOS ONE* for specific examples.	The author(s) received no specific funding for this work.

Powered by Editorial Manager® and ProduXion Manager® from Aries Systems Corporation

This statement is required for submission and **will appear in the published article** if the submission is accepted. Please make sure it is accurate.<br><br>**Funded studies**<br>Enter a statement with the following details:<br>• Initials of the authors who received each award<br>• Grant numbers awarded to each author<br>• The full name of each funder<br>• URL of each funder website<br>• Did the sponsors or funders play any role in the study design, data collection and analysis, decision to publish, or preparation of the manuscript?<br><br>Did you receive funding for this work?	
**Competing Interests**<br><br>Use the instructions below to enter a competing interest statement for this submission. On behalf of all authors, disclose any competing interests that could be perceived to bias this work—acknowledging all financial support and any other relevant financial or non-financial competing interests.<br><br>This statement is required for submission and **will appear in the published article** if the submission is accepted. Please make sure it is accurate and that any funding sources listed in your Funding Information later in the submission form are also declared in your Financial Disclosure statement.<br><br>View published research articles from *PLOS ONE* for specific examples.	The authors have declared that no competing interests exist.

**NO authors have competing interests**<br><br>Enter: *The authors have declared that no competing interests exist.*<br><br>**Authors with competing interests**<br><br>Enter competing interest details beginning with this statement:<br><br>*I have read the journal's policy and the authors of this manuscript have the following competing interests: [insert competing interests here]*<br><br>* typeset	
**Ethics Statement**<br><br>Enter an ethics statement for this submission. This statement is required if the study involved:<br><br>• Human participants<br>• Human specimens or tissue<br>• Vertebrate animals or cephalopods<br>• Vertebrate embryos or tissues<br>• Field research<br><br>Write "N/A" if the submission does not require an ethics statement.<br><br>General guidance is provided below. Consult the submission guidelines for detailed instructions. **Make sure that all information entered here is included in the Methods section of the manuscript.**	This study has been approved by the Faculty Ethics Review Committee (FERC), Faculty of Medicine, UiTM, Reference: 100 - FPR (PT.9/19) (FERC-05-23-03). Written consent was obtained prior to the study.

Powered by Editorial Manager® and ProduXion Manager® from Aries Systems Corporation

**Format for specific study types**<br><br>**Human Subject Research (involving human participants and/or tissue)**<br>• Give the name of the institutional review board or ethics committee that approved the study<br>• Include the approval number and/or a statement indicating approval of this research<br>• Indicate the form of consent obtained (written/oral) or the reason that consent was not obtained (e.g. the data were analyzed anonymously)<br><br>**Animal Research (involving vertebrate animals, embryos or tissues)**<br>• Provide the name of the Institutional Animal Care and Use Committee (IACUC) or other relevant ethics board that reviewed the study protocol, and indicate whether they approved this research or granted a formal waiver of ethical approval<br>• Include an approval number if one was obtained<br>• If the study involved *non-human primates*, add *additional details* about animal welfare and steps taken to ameliorate suffering<br>• If anesthesia, euthanasia, or any kind of animal sacrifice is part of the study, include briefly which substances and/or methods were applied<br><br>**Field Research**<br><br>Include the following details if this study involves the collection of plant, animal, or other materials from a natural setting:<br>• Field permit number<br>• Name of the institution or relevant body that granted permission	
**Data Availability**<br><br>Authors are required to make all data underlying the findings described fully available, without restriction, and from the time of publication. PLOS allows rare exceptions to address legal and ethical concerns. See the PLOS Data Policy and FAQ for detailed information.	Yes - all data are fully available without restriction

A Data Availability Statement describing where the data can be found is required at submission. Your answers to this question constitute the Data Availability Statement and **will be published in the article**, if accepted.<br><br>**Important:** Stating 'data available on request from the author' is not sufficient. If your data are only available upon request, select 'No' for the first question and explain your exceptional situation in the text box.<br><br>Do the authors confirm that all data underlying the findings described in their manuscript are fully available without restriction?	
**Describe where the data may be found in full sentences. If you are copying our sample text, replace any instances of XXX with the appropriate details.**<br><br>• If the data are **held or will be held in a public repository**, include URLs, accession numbers or DOIs. If this information will only be available after acceptance, indicate this by ticking the box below. For example: *All XXX files are available from the XXX database (accession number(s) XXX, XXX.)*.<br>• If the data are all contained **within the manuscript and/or Supporting Information files**, enter the following: *All relevant data are within the manuscript and its Supporting Information files.*<br>• If neither of these applies but you are able to provide **details of access elsewhere**, with or without limitations, please do so. For example:<br><br>*Data cannot be shared publicly because of [XXX]. Data are available from the XXX Institutional Data Access / Ethics Committee (contact via XXX) for researchers who meet the criteria for access to confidential data.*<br><br>*The data underlying the results presented in the study are available from (include the name of the third party*	All relevant data are within the manuscript and its Supporting Information files.

and contact information or URL).<br>• This text is appropriate if the data are owned by a third party and authors do not have permission to share the data.<br><br>* typeset	
Additional data availability information:	

Dr Nik Munirah Nasir

Department of Primary Care Medicine,

Faculty of Medicine,

Universiti Teknologi MARA.

5 August 2024

Dear Editor,

We wish to submit an original research article entitled "The Adaptation and Validation of the Asthma Diary Usability Questionnaire (ADU-Q) in Adults with asthma" for consideration by PLOS ONE.

We confirm that neither the manuscript nor any parts of its content are currently under consideration or published in another journal. All authors have approved the manuscript and agree with its submission to PLOS ONE.

The findings reported in this manuscript are based on the parent study looking into the Adaptation and Validation of the Asthma Self-Management Tool Usability Questionnaire (ASTHMASEM-UQ) which include both **asthma action plan** and **asthma diary**. In this paper, we report on the process of adaptation and validation of the Asthma Diary Usability Questionnaire (ADU-Q). Based on this study, ADU-Q is a valid, reliable and stable tool for assessing the usability of an asthma diary among adults with asthma. As respiratory guidelines worldwide have recommended the usage of self-management tools to improve asthma control, a valid and reliable tool to assess the usability of these self-management tool, in this case, the asthma action plan, is essential.

As asthma is one of the chronic respiratory diseases with significant morbidity and mortality, we hope to be able to contribute our findings to your readers worldwide.

We have also submitted another manuscript titled "Adaptation and validation of the Asthma Action Plan Usability Questionnaire (AAPU-Q) among adults with asthma" reporting on the findings of the validity of the developed **asthma action plan** usability questionnaire.

We would like to suggest Mohammed A. Almeshari as the academic editor in view of his stated sections and classifications which include respiratory disorders including Asthma.

We have no conflicts of interest to disclose.

Thank you for your consideration of this manuscript.

Sincerely,

Dr Nik Munirah Nasir

Manuscript

Click here to access/download;Manuscript;Asthma Diary Usability Questionnaire.docx

The adaptation and validation of the Asthma Diary Usability Questionnaire (ADU-Q) in adults with asthma

Sharifah Idayu Sayid Abdullah[1.2], Nik Munirah Nik Mohd Nasir*[1.2], Lina Lohshini Kanoo[1,2] and Nur Amirah Shibraumalisi

[1]*Primary Care Medicine Department, Faculty of Medicine, Universiti Teknologi MARA, Sungai Buloh 47000, Malaysia*

[2] *Hospital Al Sultan Abdullah (HASA), Universiti Teknologi MARA, 42300 Puncak Alam*

* Corresponding author: Nik Munirah Nik Mohd Nasir

E-mail: nikmunirah@uitm.edu.my (NM)

Author Contributions

Conceptualization: Sharifah Idayu Sayid Abdullah, Nik Munirah Nik Mohd Nasir

Data curation: Sharifah Idayu Sayid Abdullah

Formal analysis: Sharifah Idayu Sayid Abdullah

Investigation: Sharifah Idayu Sayid Abdullah

Methodology: Sharifah Idayu Sayid Abdullah[1], Nik Munirah Nik Mohd Nasir, Lina Lohshini Kanoo, Nur Amirah Shibraumalisi

Supervision: Nik Munirah Nik Mohd Nasir, Lina Lohshini Kanoo

Writing – original draft: Sharifah Idayu Sayid Abdullah

Writing – review & editing: Sharifah Idayu Sayid Abdullah[1], Nik Munirah Nik Mohd Nasir, Lina Lohshini Kanoo, Nur Amirah Shibraumalisi

Abstract

The usability assessment of an asthma self-management tool is important to ensure its effectiveness; however, the availability of a validated tool is scarce. The aim of this study is to adapt, validate and assess the reliability of the Asthma Diary Usability Questionnaire (ADU-Q) to measure the usability of an asthma diary among adult patients with asthma. The study was conducted in 3 phases. The first phase was divided into adaptation and content validation stages. The adapted questionnaires underwent domain validation by 3 experts and item content validation by 7 experts. The second phase involved face validation with 10 asthma patients who attended the primary care and respiratory clinic. This is followed by construct validation and test-retest reliability. 115 asthma patients attending the primary care and respiratory clinic were recruited to answer the ADU-Q questionnaire and 62 patients were called back to fill in the same questionnaire after 2 weeks. The psychometric properties were determined using external factor analysis (EFA) with promax rotation. The reliability testing was performed using Cronbach's Alpha and the intraclass correlation coefficient (ICC). Each item in ADU-Q went through two rounds of domain and item validation. The calculated item content validity index (I-CVI) results were >0.9. The item face validity index (I-FVI) result ranged from 0.9 to 1.0. The EFA with 3 fixed factor solutions produced 7 items loaded to Factor 1, 4 items loaded to Factor 2 and 5 items loaded to Factor 3. The Cronbach Alpha for ADU-Q was 0.982, indicating good internal consistency and the ICC value was 0.988, indicating good test-retest reliability. Based on these findings, ADU-Q is a valid, reliable and stable tool for assessing the usability of an asthma diary among adults with asthma.

1 Introduction

Poorly controlled asthma has been strongly associated with a higher mortality rate and an increase in healthcare burden. According to the RESONANCE study conducted in France, patients with uncontrolled asthma are two times more likely to be at risk of death compared to those with controlled asthma (1). In terms of healthcare burden, the cost to treat patients with uncontrolled asthma is higher than those with controlled asthma. This is reflected by comparing the costs of urgent care and maintenance care in Malaysia, where approximately USD 68 per patient was spent for urgent care compared to only USD 40 per patient for maintenance care (2).

To address the marked increase in prevalence and asthma deaths, asthma management guidelines were developed in the 1980's and 1990's. The guidelines highlighted the importance of patient empowerment and self-management which include asthma diary along with pharmacological treatment (3). Asthma diaries have been used for years as a self-management tool for asthma patients, ranging from pediatric to elderly patients. It is also used as one of the tools to assist in asthma diagnosis (4). Over the years, the method of keeping an asthma diary has evolved from paper-based to web-based (5), SMS-based (6), smartphone-based, and even linked to the usage of an inhaler device (7, 8). Recently, documentation of asthma symptoms has even been suggested to be divided into daytime and nighttime (9).

The '~~Buku Urus Kendiri Asma~~' (Asthma Self-Management Booklet) was developed by Abdul Razak, S. et al in 2016 (refer S1 appendix), which has been further refined in 2023 has been used among patients attending follow-up for asthma in a university primary care clinic. This booklet contains several sections which include patient education, clinic monitoring information, asthma diary and asthma action plan. The asthma dairy in this book is divided into

4 boxes representing 4 weeks in a month. For each box, there is daytime and nighttime column for patient to record their symptom as per S1 appendix. The purpose of the booklet is to educate and empower patients to take charge of their asthma control.

Usability assessment of the patient self-management tools is important to ensure that the tool is effective and efficient for the patient to use (10). According to the International Organization for Standardization (ISO), usability is defined as "the extent to which a product can be used to achieve specific goals with the effectiveness, efficiency, and satisfaction in a specified context of use" (11). Usability assessment is important as it has been proven to help in identifying users' issues, improving interventions to become more user-friendly, improving adherence, and reducing medical errors (12-14). One of the established models for usability is the Nielsen Usability Model, which was created by Jakob Nielsen, a leading figure in the usability field. This model identified five attributes of usability which are efficiency, learnability, satisfaction, errors and memorability (15). Learnability was defined as how easily a novice user learns about the system. Efficiency assesses how fast and easy a user performs a task after learning how to use it. The error domain assesses the ability of a product to reduce errors made by the user and how easily these errors can be corrected. The satisfaction domain assesses how pleasurable and comforting it is for the users to use the product. Memorability was defined by how easily each function of the product is remembered by the user (15).

Currently, there are questionnaires available to assess the usability of a tool; however, the items included in the questionnaire do not address the unique elements of the asthma self-management domain, which is predominantly based on the concept of Patient-Reported Outcomes (PRO) (9). Therefore, there is a need to develop a usability questionnaire that addresses specific elements in the asthma diary, as it will aid clinicians in identifying specific

usability issues among asthma patients. This study aims to adapt and validate a usability questionnaire for the asthma diary among adult asthma patients and its reliability.

2 Materials and Method

2.1 Study design and population

This study was conducted in three phases. The first phase was divided into two stages: adaptation and content validation. The second phase was face validation, while the third phase was construct validation and psychometric evaluation. The study population consisted of adult asthma patients who attended a university-based primary care clinic and respiratory clinic. The conduct of the study was based on the guidelines by the Malaysia Institute of Clinical Research (16) and the Consensus-based Standard for the selection of health Measurement Instruments (COSMIN) guidelines and principles of good practice for the validation of a questionnaire (17). The flow of the study, as illustrated in Figure 1 below:

Figure 1: Study flowchart

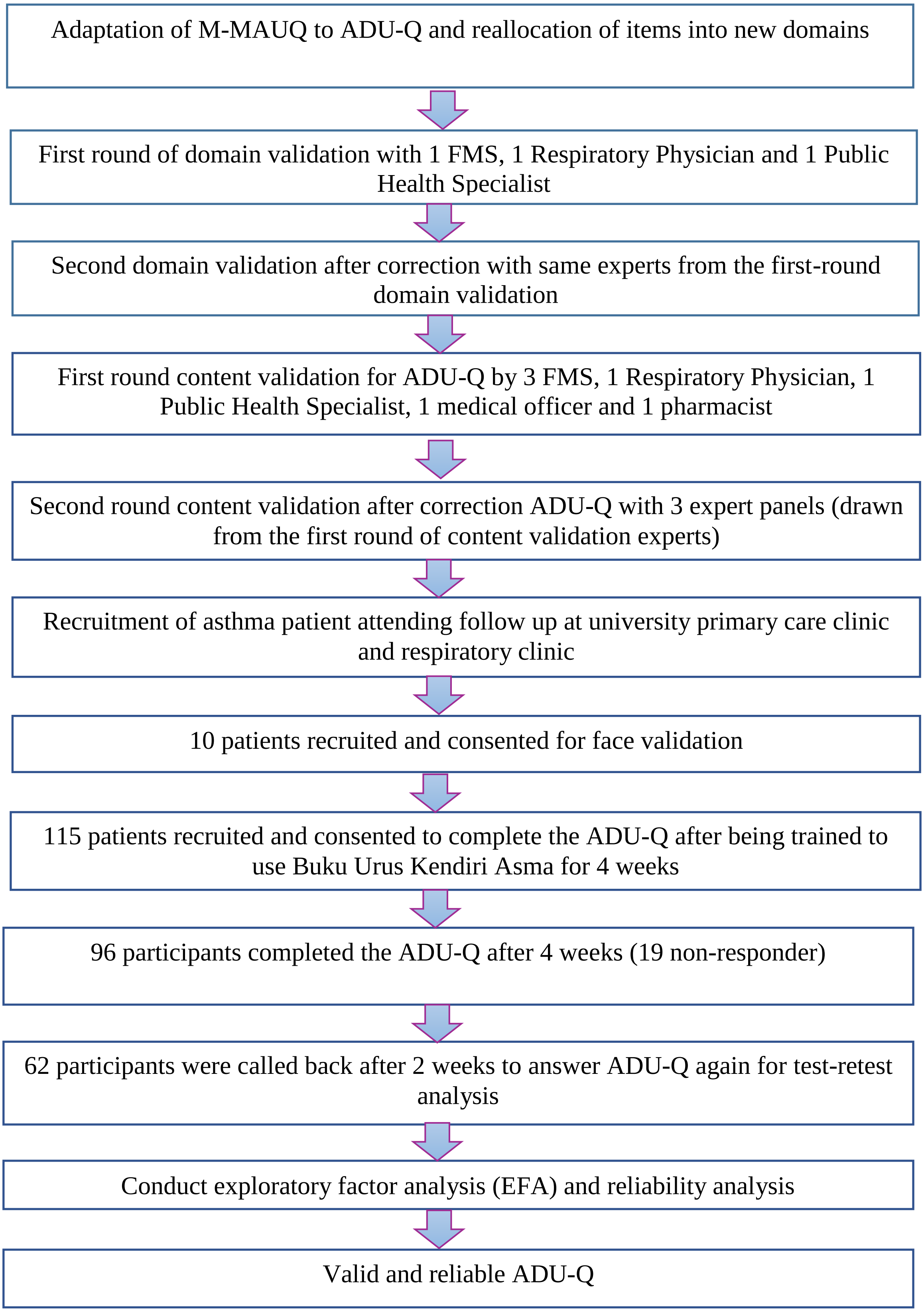

2.2 ~~The~~ study tool

The mHealth App Usability Questionnaire, or MAUQ, was developed by Zhou et al in 2019 to assess the usability of the mHealth apps (18). There are four versions of MAUQ, and each version aims to evaluate mHealth usability for different users (e.g., patients and healthcare providers), and other mHealth interaction modes (e.g., standalone, and interactive) (18). It has 18 items and is divided into 3 domains. The three main domains in MAUQ standalone (patient version) are: “Ease of use” (5 items), “Interface and satisfaction” (7 items) and “Usefulness” (6 items). The score is calculated using a 7-point Likert scale, which ranges from 1 (strongly disagree) to 7 (strongly agree). The Cronbach’s Alpha for MAUQ was 0.914 (18). The Malay language ~~translated~~ MAUQ standalone (M-MAUQ) also has ~~a~~ good reliability with Cronbach Alpha of > 0.90 (26). The M-MAUQ has a similar number of items to the original MAUQ but with no allocated domains. The score is calculated by summing all items and measuring the average score. The higher the average score, the better the usability with an average score below 4 indicating low usability (19). Permission to adapt the M-MAUQ into the Asthma Diary Usability Questionnaire (ADU-Q) was received from both the original MAUQ and M-MAUQ authors.

2.3 Phase 1: Adaptation and content validation

2.3.1 Adaptation

The original M-MAUQ underwent a review by the research team to adapt the terminology used in each item to what is relevant to the asthma diary. As the original M-MAUQ is used to assess mHealth apps, all terminologies related to app usage have been changed to hardcopy asthma diary-related terminologies and irrelevant items have been removed. Items specific to an app interface were either rephrased to reflect the usage of the hardcopy asthma diary or

dropped if it was deemed irrelevant. The adapted items are then reallocated into four new domains based on the Nielsen usability model which are Learnability, Efficiency, Satisfaction and Error. The ‘Memorability’ domain was not included as what defines this domain does not match the purpose of using the asthma diary which is to record asthma symptoms only. There are no specific functions in the diary that require the user to memorise it before it can be used.

2.3.2 Content validation

Content validation was conducted in two stages. The first stage was domain validation where content experts assess the relevance of the reallocated items under their respective new domains. According to the literature, the acceptable number of experts required for content validation is at least two (20). For this stage, 3 experts consisting of 1 Family Medicine Specialist, 1 Public Health Specialist and 1 Respiratory Physician were invited. The experts were provided with the definition of the four usability domains, the original M-MAUQ and the adapted ADU-Q questionnaire for review.

The second stage was item validation where content experts assessed the relevance and clarity of the items in evaluating asthma diary usability. In this stage, 7 experts consisting of 3 Family Medicine Specialists, 1 Public Health Specialist, 1 Respiratory Physician, 1 medical officer and 1 pharmacist were invited. The experts were provided with the original M-MAUQ and the adapted ADU-Q questionnaire for review. The expert panels were given a Google™ form link to record their rating for each item and provide written feedback.

The content validity was analysed using Item content validity index (I-CVI) with the experts scoring 1 (not relevant) to 4 (very relevant) for each item assessed (20). Items scoring 1 or 2 were re-categorised into the “not relevant” category with 0 points each. Items with a score of 3 or 4 were re-categorised into the “relevant” category with 1 point. When there are six or more

experts in the expert panel I-CVI of 0.83 or higher is considered to have good content validity (20). The scale level content validity index based on the average method (S-CVI/Ave) was computed to evaluate the relevance of the questionnaire. S-CVI/Ave is the average of the I-CVI scores for all items. The experts were also asked to offer suggestions for improvement of the items. Based on the calculated I-CVI of the expert panel and their feedback, the items that were deemed irrelevant or unclear were revised, modified or excluded as appropriate according to the expert panel's suggestions. Once any modification was made to the items, they were presented again to the expert panel. This process was repeated until a consensus was reached among the expert panel on the final items of the ADU-Q.

2.4 Phase 2: Face validation

Face validation of the revised ADU-Q from Phase 1 was then conducted among the asthma patients. A minimum of 10 sample sizes are required to perform face validity (21). Purposive sampling was used to select 10 participants who fulfilled the inclusion and exclusion criteria. The inclusion criteria were as follows: (a) Diagnosed with bronchial asthma; (b) Age more than 18 years old; (c) Has been attending the primary care clinic or respiratory clinic for follow-up for at least 6 months; (d) Has received and used the Asthma Self-Management Booklet© for at least 4 weeks; (e) able to read Malay language and (f) having the level of asthma control at Global initiative For Asthma (GINA) step 3 and below. The exclusion criteria include: (a) Have any form of cognitive impairment or mental disorder which may impact one's capability in giving informed consent; (b) Have physical disability which may impact the ability to write or read and (c) Asthma with other concurrent lung diseases.

The participants were given a Google™ form link for the ADU-Q. We asked the participants to rate each item based on the clarity and understandability of each item using a rating system 1 to 4 (1= item not clear and not understandable, 2= item somewhat clear and understandable, 3= item clear and understandable, 4= item very clear and understandable). The item level face validity index (I-FVI) was computed for each item by dichotomizing the 4-point scale. Items scoring 1 or 2 were re-categorised to “not clear and understandable” with 0 points. Items scoring 3 or 4 were re-categorized to “clear and understandable” with 1 point. The points (0 or 1) for each item were summed, and the total points were divided by the total number of respondents. An I-FVI value of at least 0.80 determined that the items were clear and well-understood (21). For items scored 1 and 2, the participants were encouraged to write down their feedback for the lack of clarity of the item. The items in ADU-Q were further refined before undergoing the psychometric evaluation.

2.5 Phase 3: Field testing and psychometric evaluation

In this phase, the psychometric evaluation of the questionnaire was assessed by conducting a cross-sectional study involving asthma patients attending the primary care clinic and respiratory clinic. The inclusion and exclusion criteria were similar to Phase 2. However, participants previously involved in Phase 2 were excluded from participating in Phase 3 of this study.

2.5.1 Sample size determination

The sample size for the final phase of ADU-Q was calculated based on the Sample to Variation ratio (SVR) of 6:1 (22). The questionnaire contains 16 items, thus the minimum sample size required was 96 participants. Considering the 20% of the non-response rate, the total sample size required was 115.

2.5.2 Sampling method, patient recruitment and data collection

The recruitment of asthma patients was done via convenience sampling from the 2nd of January until the 30th of April 2024 in the primary care clinic and respiratory clinic in a university hospital. The same researcher was assigned for data collection tasks throughout the period. Patients were approached on the day of their clinic appointment and were invited to participate in the study. A copy of the Asthma Self-Management Booklet and an explanation regarding the usage of the book were given to the patient. Patients who were interested in participating were provided with a study information sheet. The information sheet contains important information about the study such as the background, objective, benefit, study protocol, confidentiality status and contact information. For those who agreed to participate in the study, written consent was taken, and permission to contact the patient after 4 weeks to complete the questionnaire via Google™ form was obtained along with the patient's contact number.

After 4 weeks of using the ~~Buku Urus Kendiri Asma (~~Asthma Self-Management Booklet©), the patient was contacted via a phone call and screened for the inclusion and exclusion criteria to determine the eligibility for the study by direct clarification with the patient. Patients who fulfilled the eligibility criteria were given a Google™ form link via SMS for ADU-Q to be self-administered. The participants were taught with clear verbal instructions on how to fill up the questionnaire and were reminded to complete the questionnaire within 15 minutes without referring to notes or other people. The participants were free to ask for any clarification or inquiry from the researcher. Upon completing the questionnaire, the participants were requested to submit the questionnaires to the researcher by clicking the "Send" button. The

questionnaires were checked for completion by the researcher. If there were any incomplete responses, the participants were prompted to give a response if they agreed to do so.

2.5.3 Data Collection for Test and Retest

The sample size for test-retest reliability is calculated using Bonet formula giving a minimum sample size of 51 (23, 24). Considering 20% of the non-response rate, the total sample size required for test-retest reliability is 62. The participants for test and retest reliability testing were chosen via convenience sampling and contacted again via phone call 2 weeks after they had filled in the questionnaires for the first time. The same link for the questionnaires was provided to the participants for them to answer the same questionnaires.

2.6 Statistical Analysis

Data entry and statistical analysis were performed using the latest IBM SPSS Statistics Program Version 29. The psychometric properties of ADU-Q were evaluated using External Factor Analysis and Reliability Analysis.

2.6.1 Step 1 EFA: Assessment of data suitability

The data was assessed for its suitability before factor analysis was performed. The suitability of the data was analysed via the Kaiser-Meyer-Olkin test (KMO), Bartlett's Test of Sphericity and anti-image matrix. The data was said to be adequate and suitable for analysis if the KMO value > 0.5, Bartlett's test p-value <0.05 and anti-image diagonal value >0.5 (25).

2.6.2 Step 2: Factor extraction, retention and rotation

The number of factors to extract and retain was determined using a fixed number of factors method, total variance explained, scree plot and Monte Carlo parallel analysis. The fixed number of factors method was used as the domain of the items was preset before the factor analysis. By using dimensional reduction for factor, under the extraction setting, the analysis of the data was set to a correlation matrix, the fixed numbers of factors to extract were set to the number of finalized domains in the first phase and the display was set to unrotated factor solution and scree plot.

For the Monte Carlo parallel analysis, the data was analysed using SPSS and syntax. Random data was generated using syntax and compared with the real data on SPSS. After factor extraction and retention analysis, the data was rotated using promax rotation with the kappa value set to 4. Promax rotation was used as the domains were presumed to be related to each other. Due to the small sample size, the significant factor loading was set to 0.6. Afthanorhan et.al highlighted the impact of sample size on factor loading and suggested using guidelines on significant factor loading based on sample size proposed by Hair et.al in 2019 (26). A factor loading value of > 0.7 indicates that the variance of the item is sufficiently captured by the factor (27).

2.6.3 Reliability analysis

The reliability analysis of ADU-Q was analysed using Cronbach α coefficient, corrected item total correlation (CITC) and intraclass correlation coefficient (ICC). For internal consistency, Cronbach α coefficient and CITC were used to assess the internal consistency of ADU-Q. A minimum Cronbach's α coefficient value of 0.7 and CITC value of > 0.5 indicate that the questionnaire has good internal consistency (28). ICC was used for test-retest reliability analysis. The ICC value was calculated using two-way random and absolute agreement. ICC

value between 0.4 to 0.75 is considered as having good reliability and above 0.75 as having excellent reliability (29).

3 Results

3.1 Phase 1: Adaptation and Content Validation

3.1.1 Adaptation

In the adaptation stage, 11 items from M-MAUQ went through rewording, where the word app, “aplikasi” was replaced with asthma diary, “diari asma” and 5 items went through sentence rephrasing to address asthma diary usage. Items 8 and 17 (refer S2 appendix) from M-MAUQ were dropped by the research team as both items were more relevant to app-based function instead of the hardcopy booklet. Total number of items after the adaptation stage was 16. The reallocation of the items to the four domains resulted in 3 items under Learnability domain, 4 items under Efficiency domain, 8 items under Satisfaction domain and 1 item under Error domain.

3.1.2 Content validation

In the domain validation stage, all three experts agreed to move item 5 to the satisfaction domain. In contrast, two experts concurred that item 11 should fall under the efficiency domain. Thus, both items were moved as per suggestion by the experts. All three experts agreed to combine the error domain with the learnability domain, as item 16 is the only item under the error domain. Thus, item 16 was moved under the learnability domain, and the domain was renamed to Learnability and Error. After two rounds of domain validation, there were 3 final domains and the I-CVI was 1 for all items in ADU-Q.

In the item validation stage, 15 out of 16 items achieved an I-CVI of 1. Items 4 and 5 attained an I-CVI of 1, but two experts suggested adding examples to the item to make it clearer. One

expert suggested adding an alternative word for access, "akses" to the sentence in Item 8. The experts commented that all items are relevant to assess the usability of an asthma diary. Following the corrections, the items underwent a second round of item validation. After the second round of item validation, the experts retained all 16 items for ADU-Q, with the calculated S-CVI/Ave reaching 1.0. Table 1 displays the I-CVI scores for the second stage of content validation for ADU-Q.

Table 1: Final round content validity index (second stage) for item validation for ADU-Q

Item	Experts							Experts in Agreement	I-CVI
	E1	E2	E3	E4	E5	E6	E7		
LE 1	4	4	4	3	4	4	4	7	1.0
LE 2	4	4	4	4	4	4	4	7	1.0
LE 3	4	4	4	4	3	4	4	7	1.0
LE 4	4	3	4	4	4	4	3	7	1.0
EF 5	4	4	4	4	4	4	4	7	1.0
EF 6	4	4	4	4	4	3	4	7	1.0
EF 7	4	4	4	4	4	4	4	7	1.0
EF 8	4	4	4	3	4	4	4	7	1.0
EF 9	4	4	4	4	4	4	4	7	1.0
SAT 10	4	4	4	4	4	4	4	7	1.0
SAT 11	4	4	4	4	4	4	4	7	1.0
SAT 12	4	3	4	4	4	4	4	7	1.0
SAT 13	4	3	4	4	4	4	4	7	1.0

SAT 14	4	4	4	4	3	4	4	7	1.0
SAT 15	4	4	4	4	4	4	4	7	1.0
SAT 16	4	4	4	4	4	4	4	7	1.0
Content validity index average (S-CVI/Ave)									1.0

E = Experts   I-CVI = Item content validity index

LE = Learnability and error domain   EF = Efficiency domain SAT = Satisfaction domain

3.2 Phase 2: Face validation of ADU-Q

The majority of the participants score the items between 3 and 4. Five participants stated that the items were clear and simple to answer. There was one participant who scored 2 for items 14 and 15 but commented that all the items are clear and easy to understand. The calculated I-FVI for both questionnaires ranged between 0.9 and 1.0 for each item. Thus, no amendments were made, and ADU-Q went through psychometric evaluation in Phase 3. Table 2 showcased the results of I-FVI for ADU-Q.

Table 2: Face validity index for ADU-Q

Item	Raters										Raters in Agreement	I-FVI
	R1	R2	R3	R4	R5	R6	R7	R8	R9	R10		
LE 1	4	4	3	3	4	4	4	4	3	3	10	1.0
LE 2	4	4	3	3	4	4	4	4	3	3	10	1.0
LE 3	4	4	3	3	4	4	4	4	3	3	10	1.0
LE 4	4	4	4	3	4	4	4	4	3	3	10	1.0
EF 5	4	4	4	3	4	4	4	4	3	3	10	1.0

EF 6	4	4	4	3	4	4	4	4	3	3	10	1.0
EF 7	4	4	3	3	4	4	4	4	3	3	10	1.0
EF 8	4	4	3	3	4	4	4	4	3	3	10	1.0
EF 9	4	4	3	3	4	4	4	4	3	3	10	1.0
SAT 10	4	4	3	3	4	4	4	4	3	3	10	1.0
SAT 11	4	4	3	3	4	4	4	4	3	3	10	1.0
SAT 12	4	4	3	3	4	4	4	4	3	3	10	1.0
SAT 13	4	4	3	3	4	4	4	4	3	3	10	1.0
SAT 14	4	4	4	3	4	4	4	4	2	3	9	0.9
SAT 15	4	4	3	3	4	4	4	4	2	3	9	0.9
SAT 16	4	4	4	3	4	4	4	4	3	3	10	1.0

R = rater   I-FVI = Item face validity index

LE = Learnability and error domain   EF = Efficiency domain SAT = Satisfaction domain

3.3 Phase 3: Psychometric Analysis of ADU-Q

115 patients were recruited and consented to participate in the study. After 4 weeks of using the Asthma Self-Management Booklet, “Buku Urus kendiri Asma”, only 96 participants completed the ADU-Q. The other 19 participants could not be contacted during the course of the study period despite repeated attempts hence were considered to be non-responders accounting for 16.5% non-response rate.

3.3.1 Demographic and clinical characteristic of participants

The participants' ages ranged from 23 to 82. The mean age was 52.6 (SD± 14.9). Most participants were Malay (97.9%) and female (78.1%). As for asthma, the majority of

participants were under respiratory clinic follow-up (55.2%), had asthma for more than 10 years (66.7%), were at GINA stage 3 (83.3%) and used formoterol fumarate plus budesonide as asthma inhaler (39.6%). The average asthma control test (ACT) score seen in this study was 21.2 (±3.38). The demographic and clinical characteristics of participants in this study are summarized in Table 3.

Table 3: Demographics and clinical characteristics of participants (n=96)

**Characteristics of the participants**	**Frequency, n (%)**	**Mean (±SD)**
**Age**		52.6 (±14.9)
**Gender**		
Male	21 (21.8)	
Female	75 (78.1)	
**Ethnicity**		
Malay	94 (97.9)	
Others	2 (2.1)	
**Clinic type**		
Respiratory clinic	53 (55.2)	
Primary care clinic	43 (44.8)	
**Asthma duration**		
Less than 5 years	17 (17.7)	
5 to 10 years	15 (15.6)	
More than 10 years	64 (66.7)	

**GINA**		
Stage 1<br>Stage 2<br>Stage 3	7 (7.3)<br>9 (9.4)<br>80 (83.3)	
**Asthma control test (ACT) score**		21.2 (±3.38)
**Asthma inhalers**		
Salbutamol and budesonide<br>Salbutamol and Fluticasone<br>Salmeterol + fluticasone propionate and Salbutamol<br>Formeterol fumarate + budesonide<br>Formeterol fumarate + beclametasone dipropionate	11 (11.5)<br>1 (1)<br>18 (18.8)<br>38 (39.6)<br>28 (29.2)	

3.3.2 Psychometric properties

The psychometric evaluation included a total of 96 responses. All responses were complete, and no item had a missing response, indicating good data quality. The KMO value was 0.942, the Bartlett's test of sphericity p-value of less than <0.01 and all anti-image diagonal values were >0.5 indicating that the data was suitable to be analyzed. The highest communalities were for item 2 with the value of 0.968 which means that it accounts for 96% of its variability.

All items in each questionnaire went through Principal Component Analysis with subsequent promax rotations. The scree plot elbow bent at the second, third and fourth components, indicating the retention of three factors. In the parallel Monte Carlo analysis, the Eigenvalues of only one factor surpass both the mean and 95% percentile Eigenvalues. In view

of the original MAUQ having 3 domains and supported by the findings from the scree plot, the research team decided to continue to retain 3 factors.

For factor loading, the loading values for the items ranged between 0.63 to 0.939, indicating the items strong correlation with the factor. Factor 1 with the highest cumulative variance of 79% were loaded with item 10 until 16. Factor 2 with second highest cumulative variance, 7.7%, were loaded with item 1 to 4. Factor 3 with cumulative variance of 5.2% were loaded with item 5 to 9. All items had factor loading of > 0.6, which indicates that the items are valid and can be used to measure usability of an asthma diary. Table 4 showed the loading value results for ADU-Q.

Table 4: Factor loading values ADU-Q

Item	Question	Factor 1	Factor 2	Factor 3
LE 1	It was easy for me to learn to use the asthma diary		0.910	
LE 2	The asthma diary was easy to use		0.939	
LE 3	The format of the asthma diary was easy to follow		0.928	
LE 4	Whenever I made a mistake using the asthma diary, I could correct it easily		0.909	
EF 5	The asthma diary format allowed me to record my asthma symptom easily			0.658
EF 6	The information in the asthma diary was well organized, so I could easily find the information I needed			0.827

EF 7	The amount of time involved in using the asthma diary has been fitting for me			0.770
EF 8	The asthma diary improves my access/reach to health care services (Example: providing the record of asthma symptom to the treating doctor)			0.857
EF 9	The asthma diary helped me manage my asthma effectively			0.887
SAT 10	I like the format of the asthma diary	0.630		
SAT 11	The asthma diary would be useful for my health and well-being	0.826		
SAT 12	This asthma diary has all the functions and capabilities I expected it to have (Example: recording asthma symptom at home)	0.905		
SAT 13	This asthma diary provided an acceptable way to receive health care services such as performin self-assessment.	0.929		
SAT 14	I feel comfortable using this asthma diary in social settings even with the presence of friends and family	0.914		
SAT 15	I would use this asthma diary again	0.877		
SAT 16	Overall, I am satisfied with this asthma diary	0.869		

LE = Learnability and error domain EF = Efficiency domain SAT = Satisfaction domain

3.3.3 Reliability Analysis

Reliability analysis was analyzed by assessing the internal consistency using corrected total-item correlation (CITC) and Cronbach's Alpha. The calculated CITC ranged from 0.813 to 0.911, and the overall Cronbach's Alpha was 0.982. The Cronbach Alpha for Factor 1 was 0.980, Factor 2 was 0.983 and Factor 3 was 0.968. Table 5 showed the result of the analysis.

Table 5: Total-item statistics

	Scale Mean if Item deleted	Scale Variance if Item Deleted	Corrected Item-Total Correlation	Squared Multiple Correlation	Cronbach's Alpha if Item Deleted
LE 1	83.26	326.868	0.858	0.961	0.981
LE 2	83.27	326.873	0.857	0.969	0.981
LE 3	83.33	327.446	0.855	0.944	0.981
LE 4	83.32	326.958	0.829	0.896	0.981
EF 5	83.39	328.218	0.813	0.839	0.981
EF 6	83.48	322.105	0.879	0.921	0.980
EF 7	83.59	316.665	0.904	0.944	0.980
EF 8	83.46	319.367	0.895	0.947	0.980
EF 9	83.51	318.231	0.835	0.889	0.981
SAT 10	83.50	325.916	0.876	0.886	0.980

SAT 11	83.48	321.705	0.850	0.860	0.981
SAT 12	83.48	319.873	0.911	0.957	0.980
SAT 13	83.42	319.677	0.906	0.958	0.980
SAT 14	83.59	320.054	0.858	0.880	0.981
SAT 15	83.50	317.389	0.911	0.952	0.980
SAT 16	83.42	317.004	0.901	0.944	0.980
Overall Cronbach Alpha					0.982

LE = Learnability and error domain EF = Efficiency domain SAT = Satisfaction domain

For test-retest reliability, the overall ICC value for ASTHMASEM-UD AD was 0.988, indicating good reproducibility. Individual items had ICC value ranging between 0.725 to 0.985. Table 6 illustrated the result of each item ICC value.

Table 6: ICC value ADU-Q

Item	ICC	Range
LE 1	0.958	0.926 – 0.976
LE 2	0.725	0.519 – 0.843
LE 3	0.962	0.934 – 0.979
LE 4	0.966	0.941 – 0.981
EF 5	0.881	0.792 – 0.932
EF 6	0.972	0.951 – 0.984

EF 7	0.974	0.955 – 0.985
EF 8	0.977	0.960 – 0.987
EF 9	0.975	0.956 – 0.986
SAT 10	0.985	0.973 – 0.991
SAT 11	0.979	0.963 – 0.988
SAT 12	0.977	0.959 – 0.987
SAT 13	0.971	0.950 – 0.984
SAT 14	0.947	0.907 – 0.970
SAT 15	0.941	0.958 – 0.986
SAT 16	0.964	0.936-0.979

LE = Learnability and error domain   EF = Efficiency domain SAT = Satisfaction domain

4 Discussion

A questionnaire is a tool that can be used for both quantitative and qualitative methods in assessing usability (30). It can be used to measure user satisfaction and perception and obtain data on effectiveness and efficiency (31). A good usability assessment should be able to assess a user experience after a task has been performed and reflect on the user's perception of usability (30). To the researcher's knowledge, there is no validated questionnaire to assess the usability elements of an asthma diary.

The mean average usability score for ADU-Q was 5.58 (SD ±1.19) indicating good usability which is comparable to other studies using MAUQ as a method of usability assessment. For example, a study by Chumkasian et al which adapts MAUQ and uses it to assess the usability of a mobile phone-based system to promote eye donation had an average score of 5.99 SD ±0.74 (32). Other studies that used MAUQ as a usability assessment method also have shown good usability scores. These include a study to assess the usability of LactApp among breastfeeding mothers, with a mean score of 5.9 SD ±0.88 (33) and a study among adults to assess usage of mobile apps for physical activity, with mean score of 5.6 SD ±0.8 (34).

During the adaptation process of a questionnaire, items which do not reflect the construct definition of the questionnaire can be deleted (35). By using this principle, item 8 which assesses the feedback given by an app, and item 11 which assesses an app usage during internet disconnection were not relevant when using an asthma diary booklet was removed. A similar approach has been conducted by other questionnaire adaptation studies where items have been removed as it does not reflect the construct of the adapted questionnaire (36, 37). Nevertheless, the interpretation of the usability score was not affected as the usability interpretation for M-MAUQ is based on the average score.

A good questionnaire items should be reflective on its construct definition and directly measure the variable (35). In original MAUQ, the items were allocated to 3 domains, but the development of the questionnaire was based on multiple usability models (18). In contrast, the items in M-MAUQ were not allocated to any specific domain. In view of ADU-Q was based on Nielsen usability model as its theoretical model reference, the items need to reflect the usability element that it represents. Thus, four domains were determined during the adaptation phase.

A similar approach was applied in domain validation when items 5 and 11 were moved to the domain that represented it better. For item 5 which assess user opinion on asthma diary format, all experts agreed that the item reflect the definition of satisfaction domain. In the adapted ADU-Q, item 11 assessed whether an asthma diary possesses the functions and abilities required by the user. From the domain validation, the majority of the experts felt that evaluating function and ability is indicative of task completion, which is central to the efficiency domain. Based on this reasoning, item 11 was moved from the satisfaction domain to the efficiency domain. For a domain to be valid, it needs to have a minimal number of 3 items (38) which was the reason for the reallocation of item 16 from the error domain combined with items under the learnability domain. As the items need to reflect its construct, the learnability domain was renamed to learnability and error.

In item validation, item 8 had I-CVI of 1 but an alternative word was added to the word access, “akses” to improve the clarity of the questions. An item needs to be clear to avoid misinterpretation of the item by the intended user which thus, jeopardize the validity of a questionnaire (39). A similar approach has been applied in other adaptation studies where an example was added to improve the clarity of an item (40).

Face validation results reflect whether the items appear simply relevant for the population of interest. In the face validation phase, all items in both questionnaires had a high I-FVI score (0.9 to 1.0), indicating excellent clearness and ease of understanding the questions. This is further supported by written feedback by the participant claiming that the items were straightforward to respond to. Questionnaires with poor face validity has been linked to item

misinterpretation. Sensitive and difficult items also were shown to cause emotional distress to the person who answered it (39).

In factor retention, several methods are used, including parallel analysis, the Kaiser criterion (eigenvalue > 1), and the scree plot, with the latter two being the most commonly used (41). Nevertheless, each of the methods has its flaws and advantages. Taherdoost et al (2014) in his article cited Hair et al (1995) mentioned that the majority of researchers use multiple criteria to decide on factor retention (41). In this study, the factor retention was determined using a fixed number of factors, scree plot and parallel analysis. The Kaiser criterion method was not used as the original MAUQ has went through psychometric analysis which produced three factor solution. To add to that, three domains were finalized from domain validation round. Thus, the number of factors to extract was fixed to three.

Items need to have strong correlation to the factor and this relationship can be explained using factor loading. All items in ADU-Q had a significant factor loading of more than 0.6 which indicates strong factor influence on the items (42). The construct of Factor 1 was reflective of satisfaction of usability. The construct of Factor 2 fulfilled the criteria of learnability with one item addressing error. In contrast, the construct of Factor 3 was seen to fulfil the definition of efficiency. The result of factor analysis was found to be similar to the proposed domain of the adapted questionnaire. Thus, Factor 1 was maintained as Satisfaction, Factor 2 as learnability and Error and Factor 3 as Efficiency.

The overall Cronbach Alpha for ADU-Q was 0.982, indicating good internal consistency. This is comparable to other adapted MAUQ questionnaires, which also had Cronbach Alpha value of >0.9 (32, 43, 44). Test with Cronbach Alpha of >0.7 is considered reliable and has

good inter-relatedness between items in measuring the same construct. CITC value of the items ranged between 0.813 to 0.911, indicating good scale internal coherence.

In terms of test-retest reliability, individual items ICC values ranged between 0.725 and 0.985 and the overall ICC value was > 0.9, indicating good reproducibility and stability of the score over time. This value is higher compared to another adaptation and translation MAUQ study which is 0.75 (45).

5 Strength and Limitation

This study's strengths include a high response rate of 83.4% and high-quality data with no missing responses and no cross-loading items. To the authors' knowledge, there is no questionnaire which evaluates the usability of an asthma diary, thus this study filled in the gap in literature and clinical practice for this purpose. Another strength of the study is the ADU-Q is the first Malay language usability questionnaire assessing asthma diary. Thus, it is suitable to be used to assess local asthma diary in Malaysia as most patients use these tools in the Malay language.

There are four limitations to this study. First, the result of this study cannot be generalised to other multiracial respiratory clinics or primary care clinics in Malaysia, as most of the participants in this study were Malay (97.9%) and female (78.1%). Another limitation of this study is its convenient sampling method, which may lead to the introduction of sampling bias. Third, only individuals who can read and understand Malay can use the questionnaires.

6 Conclusion

ADU-Q is a valid and reliable questionnaire to assess the usability of an asthma diary. The questionnaire has 16 items with good construct validity, high internal consistency and excellent test-retest reliability.

7 Acknowledgement

The authors wish to thank Mr Zhou L, the author of the original MAUQ and Dr Nik Shanita, the author of M-MAUQ for allowing the questionnaires to be adapted into ADU-Q.

8 Ethics approval

This study has been approved by the Faculty Ethics Review Committee (FERC), Faculty of Medicine, UiTM, Reference: 100 - FPR (PT.9/19) (FERC-05-23-03)

9 References

1. Roche N, Garcia G, de Larrard A, Cancalon C, Benard S, Perez V, et al. Real-life impact of uncontrolled severe asthma on mortality and healthcare use in adolescents and adults: findings from the retrospective, observational RESONANCE study in France. BMJ Open. 2022;12(8):e060160.
2. Lai CKW, Kim YY, Kuo SH, Spencer M, Williams AE. Cost of asthma in the Asia-Pacific region. European Respiratory Review. 2006;15(98):10-6.
3. Boulet L-P, Reddel HK, Bateman E, Pedersen S, FitzGerald JM, Byrne PM. The Global Initiative for Asthma (GINA): 25 years later. European Respiratory Journal. 2019;54(2):1900598.
4. Global Initiative of Asthma. Global Strategy for Asthma Management and Prevention (2021 update). 2021.
5. Voorend-van Bergen S, Vaessen-Verberne AA, Landstra AM, Brackel HJ, van den Berg NJ, Caudri D, et al. Monitoring childhood asthma: web-based diaries and the asthma control test. J Allergy Clin Immunol. 2014;133(6):1599-605 e2.
6. Anhoj J, Moldrup C. Feasibility of collecting diary data from asthma patients through mobile phones and SMS (short message service): response rate analysis and focus group evaluation from a pilot study. J Med Internet Res. 2004;6(4):e42.
7. Archibald MM, Scott SD. Learning from usability testing of an arts-based knowledge translation tool for parents of a child with asthma. Nurs Open. 2019;6(4):1615-25.
8. Leidy N, Patrick D, Parasuraman B, Petrillo J, Mathias SD. Can Patients with Asthma Feel Their Maintenance Inhaler Working Right Away? Reproducibility and Validity of a Weekly Diary for Assessing Perception and Satisfaction. Value in Health. 2007;10(3).
9. Gater A, Nelsen L, Coon CD, Eremenco S, O'Quinn S, Khan AH, et al. Asthma Daytime Symptom Diary (ADSD) and Asthma Nighttime Symptom Diary (ANSD):

Measurement Properties of Novel Patient-Reported Symptom Measures. J Allergy Clin Immunol Pract. 2022;10(5):1249-59.
10. Bastien JM. Usability testing: a review of some methodological and technical aspects of the method. Int J Med Inform. 2010;79(4):e18-23.
11. Bevan N, Carter J, Harker S. ISO 9241-11 Revised: What Have We Learnt About Usability Since 1998? Ant Algorithms2015. p. 143-51.
12. Hinchliffe A, Mummery WK. Applying usability testing techniques to improve a health promition website. Health Promotion Journal of Australia. 2008;19(1):29-35.
13. Voshaar T, Spinola M, Linnane P, Campanini A, Lock D, Lafratta A, et al. Comparing usability of NEXThaler((R)) with other inhaled corticosteroid/long-acting beta2-agonist fixed combination dry powder inhalers in asthma patients. J Aerosol Med Pulm Drug Deliv. 2014;27(5):363-70.
14. Fairbanks RJ, Caplan S. Poor interface design and lack of usability testing facilitate medical error. Jt Comm J Qual Saf. 2004;30(10):579-84.
15. Nielsen J. What Is Usability? : Elsevier Inc.; 2010. Available from: https://booksite.elsevier.com/samplechapters/9780123751140/02~Chapter_1.pdf.
16. Bujang MA, Hon YK, Lee KY. A Step-by-Step Guide to Questionnaire Validation Research. Malaysia2022.
17. Mokkink LB, Prinsen CA, Bouter LM, Vet HC, Terwee CB. The COnsensus-based Standards for the selection of health Measurement INstruments (COSMIN) and how to select an outcome measurement instrument. Braz J Phys Ther. 2016;20(2):105-13.
18. Zhou L, Bao J, Setiawan IMA, Saptono A, Parmanto B. The mHealth App Usability Questionnaire (MAUQ): Development and Validation Study. JMIR Mhealth Uhealth. 2019;7(4):e11500.
19. Mustafa N, Safii NS, Jaffar A, Sani NS, Mohamad MI, Abd Rahman AH, et al. Malay Version of the mHealth App Usability Questionnaire (M-MAUQ): Translation, Adaptation, and Validation Study. JMIR Mhealth Uhealth. 2021;9(2):e24457.
20. Yusoff MSB. ABC of Content Validation and Content Validity Index Calculation. Education in Medicine Journal. 2019;11(2):49-54.
21. Yusoff MSB. ABC of Response Process Validation and Face Validity Index Calculation. Education in Medicine Journal. 2019;11(3):55-61.
22. Costello AB, Osborne J. Best practices in exploratory factor analysis: four recommendations for getting the most from your analysis. Practical Assessment, Research, and Evaluation. 2005;10(7):1-9.
23. Bonett DG. Sample size requirements for estimating intraclass correlations with desired precision. Stat Med. 2002;21(9):1331-5.
24. Ariffin Wn. A web-based sample size calculator for reliability studies. Education in Medicine Journal. 2018;10(3):67-76.
25. James Cook University. Factor Analysis. Assessing Suitability of Data. Singapore2023.
26. Afthanorhan A, Aimran AN, Awang Z. Five Common Mistakes for Using Partial Least Squares Path Modeling (PLS-PM) in Management Research. Contemporary Management Research. 2020;16(4):255-78.
27. Solutions S. Comprehensive Guide to Factor Analysis 2024 [cited 2024 July, 24th]. Available from: https://www.statisticssolutions.com/free-resources/directory-of-statistical-analyses/factor-analysis/.
28. Hajjar STE. Statistical Analysis: Internal-consistency Reliability And Construct Validity. International Journal of Quantitative and Qualitative Research Methods. 2018;6(1):46-57.
29. Koo TK, Li MY. A Guideline of Selecting and Reporting Intraclass Correlation Coefficients for Reliability Research. J Chiropr Med. 2016;15(2):155-63.

30. Budiu R. Quantitative vs. Qualitative Usability Testing: Nielsen Norman Group; 2017 [cited 2024 9th July]. Available from: https://www.nngroup.com/articles/quant-vs-qual/.
31. Bevan N, Carter J, Earthy J, Geis T, Harker S. New ISO Standards for Usability, Usability Reports and Usability Measures. Human-Computer Interaction Theory, Design, Development and Practice. Lecture Notes in Computer Science2016. p. 268-78.
32. Chumkasian W, Fernandez R, Win KT, Petsoglou C, Lord H. Adaptation of the MAUQ and usability evaluation of a mobile phone–based system to promote eye donation. International Journal of Medical Informatics. 2021;151:104462.
33. Quifer-Rada P, Aguilar-Camprubí L, Padró-Arocas A, Mena-Tudela D. Evaluation of the usability and utility of LactApp, a mHealth for breastfeeding support. International Journal of Medical Informatics. 2023;180:105240.
34. Tong HL, Quiroz JC, Kocaballi AB, Ijaz K, Coiera E, Chow CK, et al. A personalized mobile app for physical activity: An experimental mixed-methods study. DIGITAL HEALTH. 2022;8:20552076221115017.
35. Korb KA. Writing Questionnaire Items 2012 [Available from: http://korbedpsych.com/R09dItems.html.
36. Sim JH, Foong CC, Pallath V, Hong W-H, Vadivelu J. A Malaysian version of learning space preferences: a validation study. International Journal of Medical Education. 2021;12:86-93.
37. Mora JNC, Silva FB, Lopez RR, Cortez REC. Design, Adaptation and Content Validity Process Of A Questionnaire: A Case Study. International Journal of Management. 2016;7(7):204-16.
38. Robinson MA. Using multi-item psychometric scales for research and practice in human resource management. Human Resource Management. 2018;57:739-50.
39. Connell J, Carlton J, Grundy A, Taylor Buck E, Keetharuth AD, Ricketts T, et al. The importance of content and face validity in instrument development: lessons learnt from service users when developing the Recovering Quality of Life measure (ReQoL). Qual Life Res. 2018;27(7):1893-902.
40. Sahar NH, Badlishah-Sham SF, Ramli AS. Adaptation and Psychometric Validation of the EMPOWER-SUSTAIN Usability Questionnaire (E-SUQ) among Patients with Metabolic Syndrome in Primary Care. Int J Environ Res Public Health. 2021;18(17).
41. Taherdoost H, Sahibuddin S, Jalaliyoon N. Exploratory Factor Analysis; Concepts and theory. Advances in Applied and Pure Mathematics. 2014;27:375-82.
42. Samuels P. Advice on Exploratory Factor Analysis2016.
43. Shan Y, Ji M, Xie W, Li R, Qian X, Zhang X, et al. Chinese Version of the Mobile Health App Usability Questionnaire: Translation, Adaptation, and Validation Study. JMIR Form Res [Internet]. 2022; 6(7):[e37933 p.].
44. Kopka M, Slagman A, Schorr C, Krampe H, Altendorf M, Balzer F, et al. German mHealth App Usability Questionnaire (G-MAUQ): Translation and Validation Study2023.
45. Meryk A, Kropshofer G, Hetzer B, Riedl D, Lehmann J, Rumpold G, et al. Implementation of daily patient-reported outcome measurements to support children with cancer. Pediatric Blood & Cancer. 2021;68(11):e29279.

Click here to access/download
Supporting Information
S1 Appendix .pdf

Click here to access/download
Supporting Information
S2 Appendix.pdf

Click here to access/download
Supporting Information
S3 appendix.pdf

Click here to access/download
Other
FERC-05-23-03_Dr Sharifah.docx (1).pdf

Dr Nik Munirah Nasir

Department of Primary Care Medicine,

Faculty of Medicine,

Universiti Teknologi MARA.

8 August 2024

Dear Editor,

Thank you for your comments and queries regarding our manuscript titled *"The adaptation and validation of the Asthma Diary Usability Questionnaire (ADU-Q) in adults with asthma".* We summarize the responses according to the queries below:

**Query**	**Response**
1. Did you obtain ethics approval for this study?	Yes. This study has received ethical approval from The Faculty Ethics Review Committee (FERC), Faculty of Medicine, Universiti Teknologi MARA. The letter of approval has been uploaded 'Other'.
2. If you prospectively recruited human participants for the study – for example, you conducted a clinical trial, distributed questionnaires, or obtained tissues, data or samples for the purposes of this study, please report in the Methods:<br>i. the day, month and year of the **start and end** of the recruitment period for this study.<br>ii. whether participants provided informed consent, and if so, what type was	i. The day, month and year of the start and end of the recruitment period has been stated in the manuscript under **2.5.2 Sampling method, patient recruitment and data collection.**<br><br>"The recruitment of asthma patients was done via convenience sampling from the 2nd of January until the 30th of April 2024 in the primary care clinic and respiratory clinic in a university hospital."<br><br>ii. The participants were provided with a patient information sheet before consenting to participate in the study. The participants were given a written consent form to be filled out and

obtained (for instance, written or verbal, and if verbal, how it was documented and witnessed). If your study included minors, state whether you obtained consent from parents or guardians. If the need for consent was waived by the ethics committee, please include this information.	submitted to the researcher before completing the questionnaire. This has been stated in the same section as below:<br><br>"Patients who were interested in participating were provided with a study information sheet. The information sheet contains important information about the study such as the background, objective, benefit, study protocol, confidentiality status and contact information. For those who agreed to participate in the study, written consent was taken, and permission to contact the patient after 4 weeks to complete the questionnaire via Google™ form was obtained along with the patient's contact number."
3. If you are reporting a retrospective study of medical records or archived samples, please report in the Methods section:<br>i. the day, month and year when the data were accessed for research purposes<br>ii. whether authors had access to information that could identify individual participants during or after data collection	Not applicable

We hope that the responses and action taken are satisfactory.

Thank you for your consideration of this manuscript.

Sincerely,

Dr Nik Munirah Nasir
