# Supplementary material for: Psychometric evaluation of the Asthma Diary Usability Questionnaire (ADU-Q): An adaptation and validation of a usability assessment tool among adults with Asthma
Source: PLoS One. 2026 Mar 9;21(3):e0343631. doi: 10.1371/journal.pone.0343631 (PMC12970881; doi:10.1371/journal.pone.0343631)
Supplement: Attachment — Submitted filename: Response letter for ADU-Q_PLOS ONE.docx [file pone.0343631.s003.docx]

Dr Nik Munirah Nasir

Department of Primary Care Medicine,

Faculty of Medicine,

Universiti Teknologi MARA.

**8 August 2024**

Dear Editor,

Thank you for your comments and queries regarding our manuscript titled *“The adaptation and validation of the Asthma Diary Usability Questionnaire (ADU-Q) in adults with asthma”.* We summarize the responses according to the queries below:

| **Query** | **Response** |
| --- | --- |
| 1. Did you obtain ethics approval for this study? | Yes. This study has received ethical approval from The Faculty Ethics Review Committee (FERC), Faculty of Medicine, Universiti Teknologi MARA. The letter of approval has been uploaded ‘Other’. |
| 2. If you prospectively recruited human participants for the study – for example, you conducted a clinical trial, distributed questionnaires, or obtained tissues, data or samples for the purposes of this study, please report in the Methods:    i. the day, month and year of the **start and end** of the recruitment period for this study.    ii. whether participants provided informed consent, and if so, what type was obtained (for instance, written or verbal, and if verbal, how it was documented and witnessed). If your study included minors, state whether you obtained consent from parents or guardians. If the need for consent was waived by the ethics committee, please include this information. | i. The day, month and year of the start and end of the recruitment period has been stated in the manuscript under  **2.5.2 Sampling method, patient recruitment and data collection.**   **“**The recruitment of asthma patients was done via convenience sampling from the 2^nd^ of January until the 30^th^ of April 2024 in the primary care clinic and respiratory clinic in a university hospital.”   ii. The participants were provided with a patient information sheet before consenting to participate in the study. The participants were given a written consent form to be filled out and submitted to the researcher before completing the questionnaire. This has been stated in the same section as below:   “Patients who were interested in participating were provided with a study information sheet. The information sheet contains important information about the study such as the background, objective, benefit, study protocol, confidentiality status and contact information. For those who agreed to participate in the study, written consent was taken, and permission to contact the patient after 4 weeks to complete the questionnaire via Google™ form was obtained along with the patient’s contact number.” |
| 3. If you are reporting a retrospective study of medical records or archived samples, please report in the Methods section: i. the day, month and year when the data were accessed for research purposes ii. whether authors had access to information that could identify individual participants during or after data collection | Not applicable |

We hope that the responses and action taken are satisfactory.

Thank you for your consideration of this manuscript.

Sincerely,

Dr Nik Munirah Nasir
