# Supplementary material for: Psychometric evaluation of the Asthma Diary Usability Questionnaire (ADU-Q): An adaptation and validation of a usability assessment tool among adults with Asthma
Source: PLoS One. 2026 Mar 9;21(3):e0343631. doi: 10.1371/journal.pone.0343631 (PMC12970881; doi:10.1371/journal.pone.0343631)
Supplement: Attachment — Submitted filename: Response to reviewers.docx [file pone.0343631.s005.docx]

**Response to reviewers**

**REVIEWER 1**

| **Comment** | **Response** |
| --- | --- |
| Figure 1 contains a lot of abbreviation. Please include footnotes or relocate to the end of the Methods section as a section summary. | Figure 1 has been shifted to the end of method section and the footnotes for the abbreviation has been added (page 15) |
| Please state the language used in ADU-Q | The language used in ADU-Q is Malay language. This has been added to line 1, page 6.  “Permission to adapt the M-MAUQ into the Asthma Diary Usability Questionnaire (ADU-Q) (Malay) was received from both the original MAUQ and M-MAUQ authors. “ |
| **Domain validity** | |
| Please explain how assessment of domain validity was conducted; i.e. rating, open-ended feedback, etc.  Please state whether any iterations to the ADU-Q after domain validity assessments were made after the first round. | The conduct of domain validity assessment has been added under Content validation section (page 7)  “The assessment of the items in domain validation was done via open ended feedback.”  Yes this has been stated under Content validation section (page 7-8)  “Corrections to the adapted ADU-Q were made by the research team based on the suggestion from the experts during first round of domain validation.” |
| Please state, in general, the years of working experience of the 3 domain validity assessors and whether they had extensive experience with asthma patients/management. | Years of experience including their experience with asthma patients/management has been added under Content validation section (page 7)  “…3 experts consisting of 1 family medicine specialist, 1 public health specialist and 1 respiratory physician with working experience of more than 5 years. The family medicine specialist and respiratory physician have experience in managing patients with various stages of asthma while the public health specialist has experience in public health and preventive medicine research for chronic diseases which includes asthma.” |
| **Item validity** | |
| Please clarify whether the study method of assessing content validity is a form a modified-Delphi or otherwise. If yes, please indicate in the manuscript | The study method for content validity is based on modified-Delphi. This has been added in the paragraph on page 8.  “For the second stage, the adapted questionnaire went through item content validation process using modified-Delphi approach by 7 experts.” |
| Please clarify and cite the cutoff S-CVI/Ave value to indicate content validity | The cutoff point and citation for the S-CVI/Ave has been added to the Content validation section, page 8.  “When there are three to five experts, the S-CVI/Ave should be 1.0 to have acceptable content validity (29).” |
| Please indicate and cite the definition of “reaching consensus” | The definition and citation for “reaching consensus” added to the Content validation section, page 9.  “Reaching consensus is defined as “generally accepted opinion or decision among a group of people” (30). In this case, all experts agree that the final items of the ADU-Q is relevant. |
| In the flow chart, the second round of content validation was only conducted by 3 out of the 7 panels. Please restate this in the paragraph: who were the three and whether the reduction of the number of panels were permissible/good practice. | This explanation has been added under the Content validation section, page 8-9.  “The number of experts was reduced to three as there were no major corrections required. This number of experts are acceptable, as based on literature, the minimum number of experts for content validation are at least two (29).” |
| **Face validation** | |
| Please omit the word asthma control and rewrite “(f) having the level of asthma control at Global initiative For Asthma (GINA) step 3 and below” to “(f) is Global initiative For Asthma (GINA) step 3 and below”. | The word asthma control has been omitted and rewrote as suggested. |
| **Factor extraction** | |
| Please elaborate and cite the decision to use the fixed number of factors method | The elaboration and citation for this decision has been added under Step 2: Factor extraction, retention and rotation section (page 12-13).  “In factor retention, many researchers use multiple criteria to decide on factor retention, which include parallel analysis, the Kaiser criterion (eigenvalue > 1), and the scree plot, with the latter two being the most commonly used (36). In this study, the factor retention was determined using parallel analysis, scree plot and fixed number of factors. Kaiser criterion method was not used as the original MAUQ has went through psychometric analysis which produced three factor solution (22). Thus, the domain of the items was preset to three as per original questionnaire before the factor analysis. Considering these two factors, and recommendation by the statistical experts, the fixed factor method was chosen.” |
| **Result** | |
| Table 1: Final round content validity index (second stage) for item validation for ADU-Q Please clarify the number of experts who participated in the final round of the content validation | The final round of content validation involve 3 experts. The initial table was based on the first round of content validation, not the final round. This has been corrected in the manuscript (page 17-18). |
| Table 3 – Please substitute the word (GINA) “Stage” to “Step” in this table and throughout the manuscript | The word stage 3 has been changed to step 3 throughout the manuscript. |
| **Discussion** | |
| Page 25, first paragraph: “The mean average usability score for ADU-Q was 5.58 (SD ±1.19)”. Please clarify where readers could find this in the results section. Please elaborate on the total score permissible for the ADU-Q and what scores mean (higher, lower, categories, if any) | This discussion part was removed as this study is assessing the validity of ADU-Q and not the usability of the asthma diary. |
| Page 25, first paragraph: “The mean average usability score for ADU-Q was 5.58 (SD ±1.19)”. Please clarify whether this reflects the usability of the ‘Buku Urus Kendiri Asma’ or something else | This discussion part was removed as this study is assessing the validity of ADU-Q and not the usability of the asthma diary. |
| Page 27, first paragraph: “In factor retention, several methods are used…”. Please consider moving this paragraph to a relevant subsection under Methods section | This has been moved to Statistical Analysis section under Step 2: Factor extraction, retention and rotation pages 12-13. |
| **Limitation** | |
| “There are four limitations to this study.” Please check and make clear whether you have listed these four limitations | The limitation paragraph has been rechecked and amended. |
| Please clarify on whether or not the small number of expert panels in the content validation stage is a potential limitation | The first round of content validation comprised of recommended number of expert panel. The I-CVI result from first round was 1.0 but with some recommendation. In view of no major changes was required that affect the item itself, number of expert reduce to three. This per say is not a limitation as the I-CVI value achieved the cutoff point for good content validity which is 1.0. |
| Methods, Results and Discussions can be re-written more concisely. I recommend touching-up the manuscript for typos i.e. “dairy (page 3)”, grammatical errors and better sentence structure, for the readers’ benefits | Thank you for the suggestion. Some areas in the methods, results and discussion have been rechecked. The manuscript has been screened again for grammatical error and has been corrected. |

**REVIEWER 2**

| **Comments** | **Response** |
| --- | --- |
| The abstract should elaborate more on the background of the study. Perhaps including the current situation on the control of asthma among the Malaysian population which further emphasize the urge to conduct this research. The content of abstract was not equally divided according to subsection. Most of the content are elaborating on the methods part. Perhaps the results and discussion parts should be refine in the abstract for the readers to get a better overview of the research. | The abstract has been added. Situation regarding asthma prevalence and control has been added in the abstract.  The methods and results section have been amended. |
| **Introduction** | |
| In the first paragraph of the introduction, is it possible to add more details (RESONANCE study, no. of patients included with age range and other details such as comorbidities). The details need not be too long, it can be summarised within one or two sentences | More details regarding RESONANCE study has been added under the Introduction section, page 3.  “According to the RESONANCE study which included individuals age 12 years and above from a large populational databases in France, patients with uncontrolled asthma are two times more likely to be at risk of death compared to those with controlled asthma (6).” |
| In terms of healthcare burden, the cost to treat patients with uncontrolled asthma is higher than those with controlled asthma'. Is this sentence referring to the Malaysian population or other studies?. The following sentence on the costs of urgent care and maintenance care in Malaysia is also confusing as the costs were stated in USD. Perhaps the costs in both currencies can be stated | This sentence mainly referred to the cost in Malaysia which was part of the result of the cost of asthma in Asia-Pacific region study.  The currency of USD has been converted into Malaysia ringgit. Both currencies were included in the sentence under Introduction section, page 3.  “In terms of healthcare burden in Malaysia, approximately RM 301 (USD 68) was spent per patient for urgent care compared to only RM 177 (USD 40) per patient for maintenance care (7).” |
| Paragraph 3 and 4 are disconnected. The use of asthma self-management booklet was developed in 2016 and there had been an updated version in 2023 indicating some precipitating issues/problems leading to the update of the booklet. This could be related to the need for usability assessment. Elaboration on this will make a better flow | The explanation regarding the booklet has been moved to ‘The study tool’ section. The problems related to the book has been included as well (page 6).  “This Asthma Self-Management Booklet was refined in 2023 after qualitative feedback from both physicians and asthma patient that have used the booklet. The refinement included improvement in the size of font and layout, asthma education content and graphics to make it more user friendly.” |
| **Methods** | |
| The author may want to spell out FMS in figure 1 and use abbreviations in the following paragraphs | Figure 1 has been shifted to the end of method section and the footnotes for the abbreviation has been added (page 15) |
| **Results** | |
| Put the reference range underneath each table for ease of reference (factor loading>0.6 indicates items validity) | The reference has been added under each table for ease of reference. |
| **Discussion** | |
| There were several definitions given that could have been mentioned in the introduction instead to allow other important points to be included in the discussion e.g. elaboration on the results | The definition of several terms have been removed to relevant sections to allow more focus discussion on the result of the study. |
| Some parts of the discussion are redundant with the results for example "Test with Cronbach Alpha of >0.7 is considered reliable" and "overall ICC value was > 0.9 indicating good reproducibility and stability of the score over time. | The relevant discussion sections have been amended to avoid redundancy. |
| To add discussion on the demographic of the participants | The discussion regarding the demographic of the participants has been added in the second paragraph (page 27-28) |
| **Strength and Limitation** | |
| In the results part it was stated that during the data collection, a copy of the Asthma Self-Management Booklet and an explanation regarding the usage of the book were given to the patient. Would there be any bias imposed to the data collection process. | The explanation is mainly about how to fill in the asthma diary. All participants were given the same Google form link and the participant can remain anonymous when answering the form to avoid the researcher from influencing the response given by the participants. |
| **Statistical Analysis** | |
| It was stated that all items in the questionnaire went through principal component analysis. Is this related to determining whether the factors are correlated before deciding to use promax rotations for analysis?. Is learnability strongly correlated with efficiency and satisfaction? etc. | The items went through principal component is to determine dimension reduction. This step is taken to compare the adapted questionnaire factor reduction and the factor produce by the original questionnaire. The promax rotation was used as according to literature, the elements of usability intercorrelated with each other. |
