# Supplementary material for: Psychometric evaluation of the Asthma Diary Usability Questionnaire (ADU-Q): An adaptation and validation of a usability assessment tool among adults with Asthma
Source: PLoS One. 2026 Mar 9;21(3):e0343631. doi: 10.1371/journal.pone.0343631 (PMC12970881; doi:10.1371/journal.pone.0343631)
Supplement: Attachment — Submitted filename: Reviewer Comments for PONE-D-24-32929R1.docx [file pone.0343631.s004.docx]

**Reviewer’s comments for PONE-D-24-32929R1**

Congratulations on producing a review of the manuscript. My comments serve to improve clarity and reader’s understanding.

**Title:**

Title clearly reflects the study scope and studied population, though slightly plain. If permitted, consider rephrasing the title. The use of a colon (:) in the title improves paper’s visibility in database searches.

**Abstract:**

Please rewrite the abstract guided by a structured format (Background 🡪 Main Objective 🡪 Methods 🡪 Results 🡪 Conclusion). An abstract should provide a concise overview of the study process whereas your abstract is a narrative and may appear too detailed. Recommend making more concise.

**Introduction:**

The background of study is sufficient to demonstrate to extent of problem, the role of asthma self-management and that a tool should be usable. However, please introduce the actual asthma diary itself as a foreshadowing to the aim of study. I suggest moving and rewriting the paragraph “The asthma diary used in this research was taken from The Asthma Self-Management Booklet…” from Methods (pg 6) to Introduction, in order to improve readers’ understanding of the study (which is to adapt and validate the M-MAUQ into the ADU-Q).

**Methods:**

If I understand correctly, the novelty of this research is the development of ADU-Q. However, the intention of the ADU-Q is first described only at the end of an extensive paragraph on M-MAUQ (pg 5). Please restructure the paragraph to begin with introducing the development of ADU-Q. In addition, please clarify whether all questions were adapted exclusively from the M-MAUQ. I suggest omitting the section “Study Tool” (pg 5) and moving contents to Phase I Adaptation and Content Validation (pg 6).

In Content Validation, “The experts were required to discuss and assess the relevance…” (pg. 7) , please clarify whether this is a form of Nominal Group Technique.

Based on the calculated I-CVI of the expert panel and their feedback, “the items that were deemed irrelevant or unclear were revised, modified or excluded as appropriate according to the expert panels’ suggestions” (pg. 8). Please clarify on how you decide whether which item gets omitted and which item gets iterated. Is there an objective measure (score/threshold level) as a guide or otherwise?

Reaching consensus is defined as “generally accepted opinion or decision among a group of people. In this case all experts agree….” (pg. 9). Please specify if there is an objective measurement of consensus e.g all items achieving a specific S-CVI or I-CVI value?

Please rephrase Figure 1 (pg. 15) concisely according to manuscript standard (poor scientific writing). Consider to alternately use concisely labeled pictograms to describe the study process instead.

**Discussion**

“The asthma diary has been part of various asthma guideline recommendation as a self-management tool for patients but a study by Tsang et al demonstrated that compliance to completing the asthma diary was low with only a quarter of participants still monitoring after six months. Thus, the lack of a suitable tool to assess the asthma diary usability has limited our ability to identify if this could be the potential reason for suboptimal compliance to improve on its usage….” (pg. 27) 🡨This is a great study justification, and should be moved from Discussion to Introduction.

“However, to ensure that the participants remain homogenous, the inclusion criteria was set at GINA step 3 and excluded those with other underlying lung diseases” (pg. 28). Please correct to “GINA Step 3 **and below**”.

Please include that reliance of patient’s self-reporting is a limitation of this study however, as usability reflects user self-reflection, self-report is the most appropriate method for the study (pg 29)

**Overall**

Well done on producing valid and substantive research and manuscript. However, I strongly recommend a thorough language edit to polish this manuscript grammatically, improve sentence structure, flow and in alignment with scientific writing style. Thank you.
